# REFLECTIONS FROM THE PERSPECTIVE OF AN ADVOCATE AND CASE MANAGER

**DOI:** 10.1093/geroni/igz038.2339

**Published:** 2019-11-08

**Authors:** Tracy Keibler

**Affiliations:** APparentPlan, Eden Prairie, Minnesota, United States

## Abstract

From her perspective as director of ApparentPlan, a nonprofit care agency to assist low income consumer of LTC, and as co-founder and director of the MN Long-Term Care Think Tank, an advocacy organization. Ms. Keibler will reflect on these findings and next steps.

